# Efficacy of Daratumumab‐Based Regimens for Extramedullary Pulmonary Plasmacytoma: A Case Report

**DOI:** 10.1002/cnr2.2149

**Published:** 2024-11-15

**Authors:** Danilo De Novellis, Pio Zeppa, Elisabetta Maffei, Valentina Giudice, Carmine Selleri, Bianca Serio

**Affiliations:** ^1^ Hematology and Transplant Center University Hospital “San Giovanni di Dio e Ruggi d'Aragona” Salerno Italy; ^2^ Department of Medicine, Surgery, and Dentistry University of Salerno Baronissi Italy; ^3^ Anatomy Pathology Unit University Hospital “San Giovanni di Dio e Ruggi d'Aragona” Salerno Italy

**Keywords:** daratumumab, extramedullary disease, multiple myeloma

## Abstract

**Introduction:**

Multiple myeloma (MM) with pulmonary extramedullary disease is rare and usually associated with poor prognosis, and no data on daratumumab‐based regimens have been reported yet.

**Case Presentation:**

Here, a 64‐year‐old man with pulmonary plasmacytoma received daratumumab‐based regimens and has achieved a very good partial response with lung mass disappearance and overall survival of 16 months. He did not receive autologous stem cell transplantation because of several comorbidities, such as severe drug‐induced neuropathy and *JAK2*‐mutated myeloproliferative neoplasm with marked splenomegaly.

**Conclusions:**

We showed the efficacy of daratumumab in combination with targeted therapies for the treatment of pulmonary MM.

## 1 Introduction

Multiple myeloma (MM) is a malignant hematological disease characterized by bone marrow (BM) proliferation of clonal plasma cells and hyperproduction of monoclonal proteins (M‐proteins) with diffuse organ damage. Neoplastic cells can also accumulate within soft tissues, termed extramedullary disease (EMD) when associated with ≥10% of BM plasma cells, and differs from plasmacytoma, as this latter is associated with clonal proliferation in tissues neighbor to the bone [1]. EMD could be present at diagnosis (primary) in 6%–8% of patients or more frequently at relapse (secondary) in up to 30% of cases [2]. EMD occurrence is associated with a poorer prognosis, regardless of the therapeutic agents used for its treatment [3]. Daratumumab, an anti‐CD38 fully humanized IgGκ monoclonal antibody, is highly effective for MM treatment and also as a first‐line approach for eligible and ineligible patients to autologous stem cell transplantation (ASCT) [4]. However, limited data on the efficacy of daratumumab for treatment of newly diagnosed MM with primary plasmacytoma or EMD. Here, we described a rare lung plasmacytoma successfully treated with daratumumab‐based regimens as first‐line therapy.

## 2 Case Presentation

**FIGURE 1:**
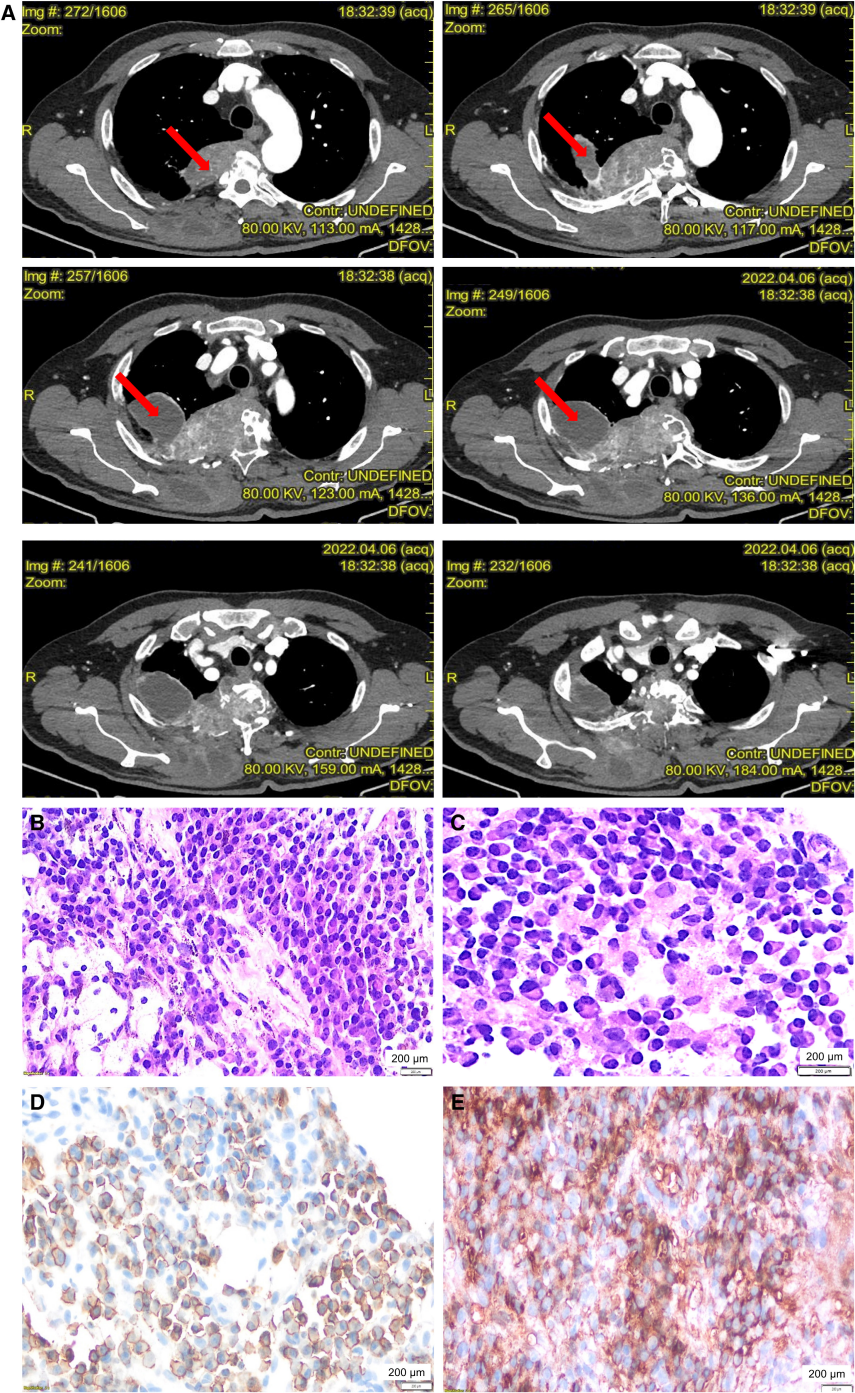
Clinical presentation at diagnosis. (A) CT scan imaging at diagnosis showed pulmonary plasmacytoma in the posterior half of the right upper pulmonary lobe, eroding vertebral somas of D2, D3, D4, and D5, the posterior arch of the fourth rib, the right transverse apophyses of D3 and D4, the right pedicle of D2, and invading the medullary canal in D2–D4 tract (red arrows). (B) Core‐needle biopsy of the lung mass displaying plasma cell proliferation, foamy histiocytes, and inflammatory cells (hematoxylin–eosin, ×270; scale bar = 200 μm). (C) Higher magnification of well‐differentiated plasma cells (hematoxylin–eosin, ×430; scale bar = 200 μm). (D) Immunohistochemistry for CD138, a plasma cell marker (positivity for CD138 is evidenced as purple staining; CD138, APAP immunostaining, ×270; scale bar = 200 μm). (E) Immunohistochemistry for light chain clonality showing a diffuse, cytoplasmic, kappa light chain restriction without lambda chain expression (positivity for kappa light chain is shown as purple staining; Kappa light chain, APAP immunostaining, ×270; scale bar = 200 μm).

In April 2022, a 64‐year‐old man presented to the Emergency Unit, University Hospital “San Giovanni di Dio e Ruggi d'Aragona,” Salerno, Italy, complaining of diffuse and intense bone pain. A whole‐body CT scan was performed, showing a large neoplastic mass (16 × 10 × 15 cm) at the right upper lobe of the lung with bone erosion of posterior arches of the fourth and fifth costa and of vertebral somas of D2, D3, D4, and D5 (Figure 1A). In addition, multiple, widespread lytic bone lesions were identified in the pelvis, femur, ribs, and vertebrae. In May 2022, a CT‐guided biopsy of the lung mass was performed, and immunohistochemistry analysis showed a diffuse proliferation of neoplastic plasma cells with light κ chain restriction, a nonspecific inflammatory pabulum, and multiple histocytes (Figure 1B). Therefore, the patient was sent to our Hematology Unit and screened for MM, displaying an absence of anemia (hemoglobin, 14 g/dL [normal range, 12–16 g/dL]) and Bence Jones proteinuria, normal albumin levels (3.8 g/dL [normal range, absent]), low calcium serum levels (8.4 mg/dL [normal range, 8.6–10.2 mg/dL]), renal impairment (glomerular filtration rate [GFR], 44 mL/min [normal range, ≥90 mL/min] with creatinine levels of 2 mg/dL [normal range, 0.8–1.2 mg/dL]), and increased β_2_‐microglobulin (4.5 mg/L [normal range, 0.80–2.34 mg/L]). Serum immunofixation electrophoresis showed a low IgG κ component (0.3 g/dL [normal range, absent]) with a balanced serum‐free light‐chain ratio. Neoplastic BM CD38^+^CD138^+^CD19^+^CD45^+/dim^ plasma cells were 5% by immunohistochemistry without high‐risk genetic abnormalities by fluorescence in situ hybridization and normal karyotype (46,XY). The revised international staging system was II. Although BM plasma cell count was relatively low, the presence of numerous lytic areas associated with lung plasmacytoma suggested a systemic active MM. Moreover, the patient suffered from polycythemia vera with V617F *JAK2* mutation, diabetes mellitus, chronic kidney disease, and chronic obstructive pulmonary disease (COPD). However, due to his young age, he was considered eligible for ASCT, and dara‐VTD induction chemotherapy was started with subcutaneous daratumumab (1800 mg weekly for the first two cycles, and then every 2 weeks for the third and fourth cycles), bortezomib (1.3 mg/m^2^ on Days 1, 4, 8, and 11), thalidomide (100 mg daily), and dexamethasone (40 mg twice weekly for the first two cycles, and then reduced to 20 mg twice weekly), according to CASSIOPEIA trial protocol [4]. Monthly intravenous zoledronic acid was tapered according to GFR, and acyclovir and trimethoprim/sulfamethoxazole were given as anti‐infectious agent prophylaxis.

**FIGURE 2:**
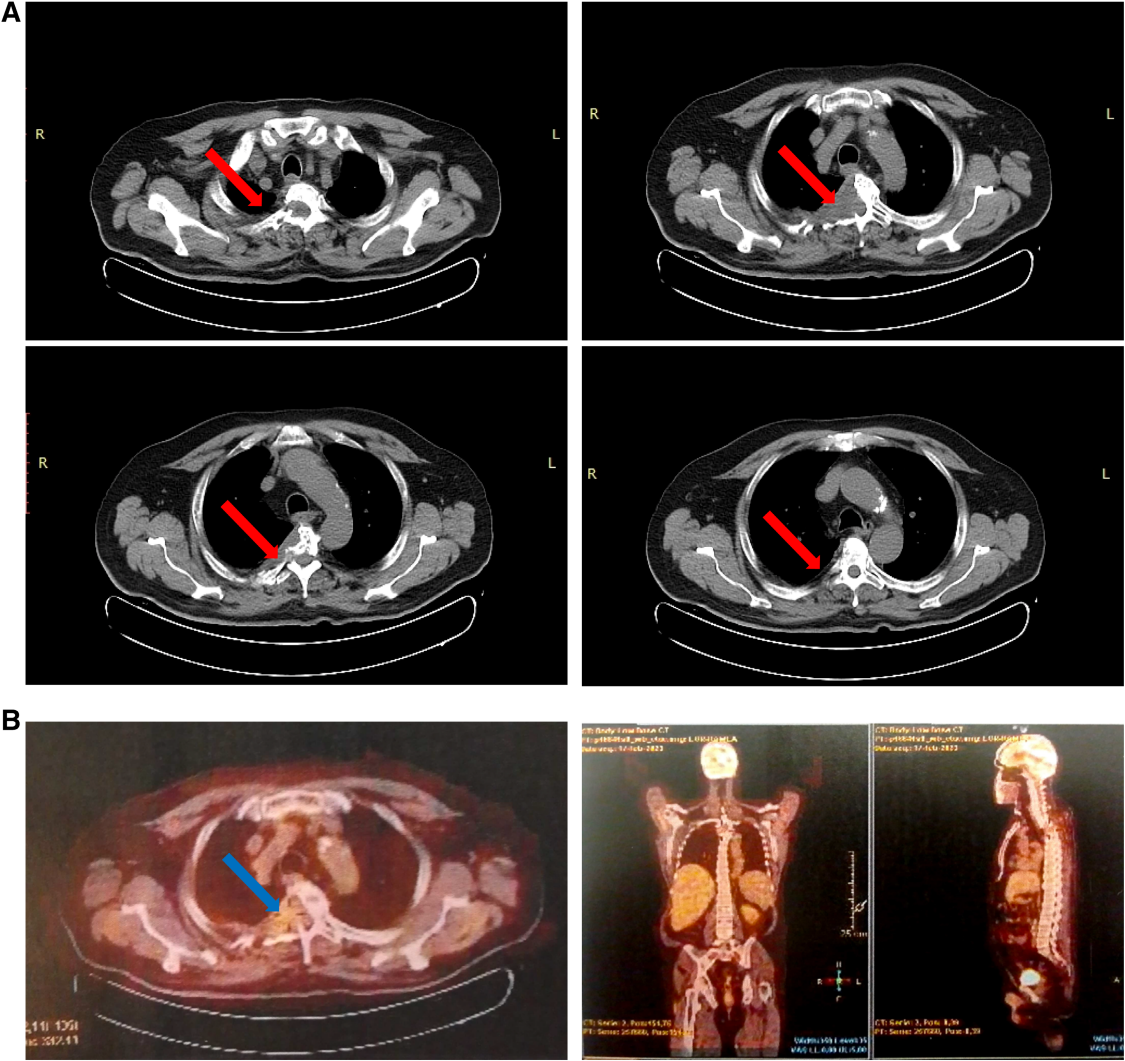
Imaging re‐evaluation. (A) CT and (B) FDG‐PET scan imaging showing a significant size reduction of pulmonary plasmacytoma (red arrows) with minimal residual uptake of radioactive marker (blue arrow).

In October 2022, after four cycles of therapy, the patient achieved a very good partial response (VGPR) with complete disappearance of bone pain; however, he developed severe bortezomib‐ and thalidomide‐related grade III sensor motor neuropathy significantly affecting daily activities and requiring analgesics, l‐acetyl‐carnitine, high‐dose pregabalin, and permanent drug discontinuation. A CT scan re‐evaluation was not performed because of patient's will. Moreover, spleen size increased (from 14 to 19 cm), COPD worsened by spirometry, and general status deteriorated, making the patient ineligible for ASCT at this point. Therefore, based on clinical status worsening and thalidomide intolerance, the dara‐RD regimen was started with daratumumab, lenalidomide, and dexamethasone, according to MAIA trial protocol for transplant‐ineligible patients [5]. In January 2023, after seven well‐tolerated cycles, an imaging re‐evaluation was carried out, showing a significant reduction in the lung mass without metabolic activity by fluoro‐18‐fluorodeoxyglucose positron emission tomography, as well as for other lytic areas, except for the D3 soma with residual metabolic activity (standardized uptake value, 2.5) (Figure 2). In November 2023, at the time of writing, after 16 months from diagnosis and 14 cycles of chemotherapy, the patient is alive and still in VGPR with improved general conditions and without BM neoplastic plasma cells and bone pain.

## 3 Discussion

In this case report, we documented an effective alternative therapeutical approach for the treatment of a rare lung plasmacytoma using daratumumab‐based regimens as first‐line therapy. Indeed, MM with primary extramedullary plasmacytoma is associated with a very poor outcome due to its aggressive phenotype, drug resistance, and short survival, even in the era of new therapeutic agents [6]. EMD is more commonly observed in the liver or lymph nodes, while lung involvement is rare (2.65% of cases) and manifested as pulmonary mass with or without unilateral pleural effusion and wheezing cough [2]. Patients with EMD have very short overall survival, ranging from 2.8 to 4 months, and frequently carry high‐risk cytogenetic abnormalities, such as t(4;14), t(14;16), gain(1q21), or del(17p) [6]. Some evidence suggests that EMD is more susceptible to novel drugs than to standard chemotherapy [7]; however, no data on the efficacy of daratumumab, a novel anti‐CD38 monoclonal antibody, are reported for pulmonary EMD treatment.

Previous studies have reported low efficacy of daratumumab monotherapy in refractory/relapsed MM with EMD, showing a poor overall response rate (ranging from 16.7% to 22%) and short progression‐free (1.4 months) and overall survival (4.6 months) [8, 9, 10]. In contrast, our patient with pulmonary EMD without high‐risk genetic abnormalities greatly benefited from daratumumab‐based regimens with bone pain relief, hematological response (VGPR with an overall survival of 16 months), and disappearance of lung plasmacytoma and lytic lesions with only minimal residual uptake on the D3 vertebral soma. Unfortunately, the patient was not eligible for ASCT because of severe bortezomib‐ and thalidomide‐induced neuropathy and *JAK2*‐mutated myeloproliferative neoplasm with marked splenomegaly. However, daratumumab‐based regimens allowed long‐term disease control.

## 4 Conclusions

In the setting of rare MM presentations, such as lung plasmacytoma, with unfavorable clinical outcomes, we showed that anti‐CD38 monoclonal antibodies in combination with targeted therapies could be an effective treatment option in patients with newly diagnosed MM with pulmonary EMD.

## Author Contributions

Conceptualization: Danilo De Novellis and Carmine Selleri. Methodology: Danilo De Novellis, Elisabetta Maffei, and Pio Zeppa. Investigation: Danilo De Novellis and Valentina Giudice. Data curation: Danilo De Novellis and Valentina Giudice. Writing – original draft preparation: Danilo De Novellis and Valentina Giudice. Writing – review and editing: Pio Zeppa and Carmine Selleri. Supervision: Carmine Selleri. All authors have read and agreed to the published version of the manuscript.

## Ethics Statement

The patient received informed consent obtained in accordance with the Declaration of Helsinki (World Medical Association 2013), and protocols were approved by the local ethic committee (Ethics Committee “Campania Sud,” Brusciano, Naples, Italy; prot./SCCE n. 24988). Written informed consent has been obtained from the patient to publish this paper.

## Conflicts of Interest

The authors declare no conflicts of interest.

## Data Availability

Data are available upon request by the authors.
